# Evaluation for Synergistic Effects by Combinations of Photodynamic Therapy (PDT) with Temoporfin (mTHPC) and Pt(II) Complexes Carboplatin, Cisplatin or Oxaliplatin in a Set of Five Human Cancer Cell Lines

**DOI:** 10.3390/ijms19103183

**Published:** 2018-10-16

**Authors:** Carsten Lange, Patrick J. Bednarski

**Affiliations:** Department of Pharmaceutical and Medicinal Chemistry, Institute of Pharmacy, University of Greifswald, Friedrich-Ludwig-Jahn-Straße 17, 17487 Greifswald, Germany; langec@uni-greifswald.de

**Keywords:** cancer therapy, Pt(II) complexes, cisplatin, carboplatin, oxaliplatin, photodynamic therapy, mTHPC, combination assay, synergism

## Abstract

The platinum(II) complexes carboplatin (CBDCA), cisplatin (CDDP) and oxaliplatin (1-OHP) are used as anticancer drugs in a large number of tumour chemotherapy regimens. Many attempts have been made to combine Pt(II)-based chemotherapy with alternative treatment strategies. One such alternative anticancer approach is known as photodynamic therapy (PDT), where a non-toxic photosensitizer (PS) produces oxidative stress via the formation of reactive oxygen species (ROS) after local illumination of the affected tissue. A very promising PS is 5,10,15,20-tetra(m-hydroxyphenyl)chlorin (mTHPC, Temoporfin), which is approved for the treatment of head and neck cancer in Europe. In the present study, a combination of mTHPC-mediated PDT and either CBDCA, CDDP, or 1-OHP was applied to five human cancer cell lines from different tumour origins. Cytotoxicity was determined by the MTT assay and synergistic effects on cytotoxicity were evaluated by calculation of Combination Indices (CI). Synergy was identified in some of the combinations, for example, with 1-OHP in three of the tested cell lines but antagonism was also observed for a number of combinations in certain cell lines. In cases of synergy, elevated ROS levels were observed after combination but apoptosis induction was not necessarily increased compared to a treatment with a single compound. Cell cycle analysis revealed a formation of apoptotic subG1 populations and S phase as well as G2/M phase arrests after combination. In conclusion, pre-treatment with mTHPC-PDT has the potential to sensitize some types of tumour cells towards Pt(II) complexes, in particular 1-OHP but synergy is highly dependent on the type of cancer.

## 1. Introduction

**Scheme 1:**
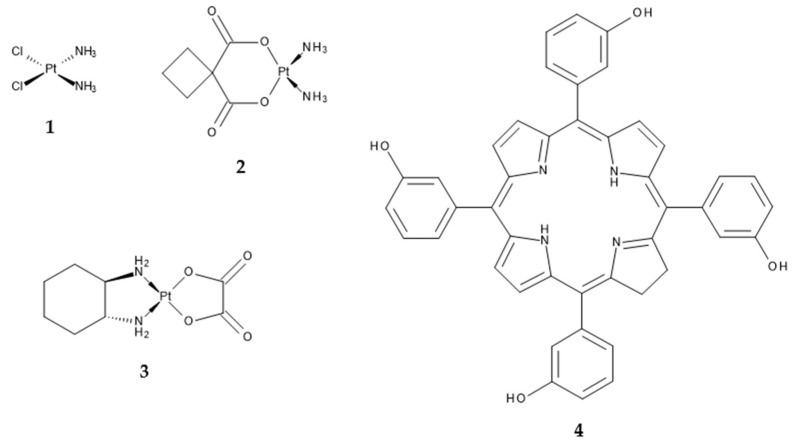
Chemical structures of the Pt(II) complexes cisplatin (**1**), carboplatin (**2**) and oxaliplatin (**3**) as well as the photosensitizer mTHPC (**4**).

The classic cancer therapy with chemotherapeutic agents is, among surgery, immunotherapy, radiation and targeted therapy, one of the central treatment strategies against neoplastic diseases. Platinum(II)-based agents are one group of anticancer drugs in chemotherapy, from which especially cisplatin (*cis*-diamminedichloridoplatinum(II), CDDP) (**1**), carboplatin (*cis*-diammine-1,1-cyclobutanedicarboxylatoplatinum(II), CBDCA) (**2**) and oxaliplatin (*trans*-*R*,*R*-cyclohexane-1,2-diamineoxalateplatinum(II), 1-OHP) (**3**) (Scheme 1) are used in a very high number of tumour chemotherapies [1,2,3,4,5,6]. All three mentioned Pt(II) complexes act via the formation of intrastrand and, to a lesser degree, interstrand DNA crosslinks after aquation and accompanied activation [4,7,8]. The DNA adducts ultimately lead to the induction of apoptosis [6]. Due to the persistence of severe adverse effects and tumour resistance against the treatment with Pt(II) complexes, many attempts have been made to combine Pt(II)-based chemotherapy with alternative treatment strategies, rather than other anticancer drugs [9,10,11]. One of those alternative anticancer approaches is known as photodynamic therapy (PDT). PDT is a non-invasive approach for the treatment of malignancies, where a non-toxic photosensitizer (PS) is activated by light of a certain wavelength to generate reactive oxygen species (ROS) within the affected tissue. The oxidative stress then leads to locally restricted cellular death in the illuminated area. PDT displays some advantages over classic cancer therapy, that is, no systemic effects due to non-existent toxicity in the absence of light, selectivity due to the local restriction of the illumination area, non-invasiveness, no carcinogenicity, no cumulative toxicity, selective retaining of the PS in tumorous tissue and effective recovery of the healthy surrounding tissue [12,13]. Disadvantages include the restricted applicability to cancers at advanced stages due to the limited penetration of light into the tumour as well as photosensitivity after the treatment as the primary side effect due to a delayed clearance of the PS. Furthermore, it has to be ensured that tumours can be illuminated externally or endoscopically, at least without losing the advantage of minimal or non-invasiveness [12]. One very promising PS is the reduced porphyrin derivative 5,10,15,20-tetra(m-hydroxyphenyl)chlorin (mTHPC, Temoporfin) (**4**) (Scheme 1), which was approved in 2001 as Foscan^®^ for the palliative treatment of head and neck cancer [14].

Enhanced cytotoxic effects have already been observed after the combination of PDT and Pt(II) complexes in some in vitro studies. These included combinations of CDDP and the photosensitizers Photofrin against oesophageal [15] and mouse lymphoma cells [16] or EtNBS-COOH against small cell lung cancer cells (SCLC) [17]. CBDCA has been combined with 9-hydroxypheophorbide α (9-HPbD) and radachlorin, respectively, for the treatment of laryngeal cancer cells [18,19,20] as well as with verteporfin against ovarian [21] or Photofrin against cervical cancer cells [22]. 1-OHP has been used in a combined approach with hypericin in colorectal cancer cells [2]. All of these combinations led to enhanced cytotoxic effects and increased anticancer efficacy in vitro. However, in all of these studies just one cancer cell line was used in the evaluations of synergy.

In the present study, we evaluated the in vitro cytotoxic and apoptotic effects of a combination of mTHPC-mediated PDT and either cisplatin, carboplatin, or oxaliplatin on five human cancer cell lines from different tissue origins (bladder, cervix, oesophagus, lung and oral cavity). The insufficient treatment of large solid tumours by PDT and persistent severe adverse effects after chemotherapy with Pt(II) complexes make the combination of both approaches interesting as it may help overcome drawbacks that each treatment modality brings on its own. Therefore, the aim of the study was to investigate whether the sensitivity of cancer cells towards Pt(II) complexes can be modified due to prior mTHPC-PDT to allow for lower doses of the chemotherapeutic agents and thus to reduce adverse effects at a consistent antitumor efficacy.

We have found that a combined treatment approach of mTHPC-PDT and Pt(II) complexes has to be evaluated individually for each specific cancer type, because combinations do not always lead to increases in the antitumor efficacy and can even be antagonistic.

## 2. Results

### 2.1. Detection of Synergism after Combination of mTHPC-PDT and a Pt(II) Complex

**Figure 1:**
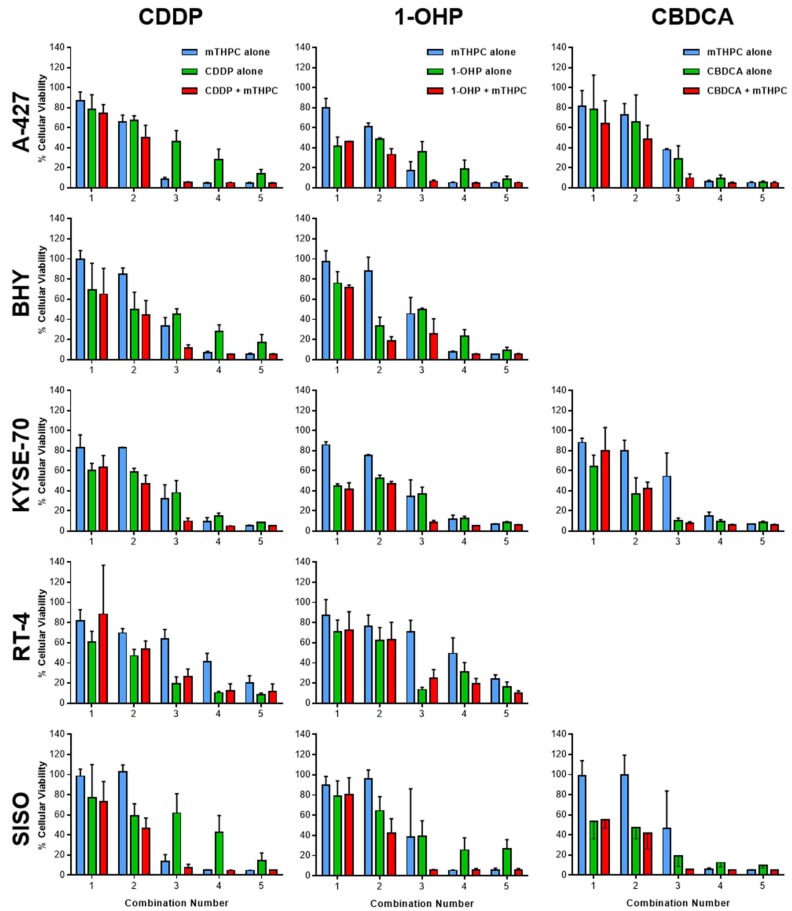
Loss of cellular viability after treatment with mTHPC (blue) or Pt(II) complex alone (green) or in combination (red) in various cell lines as assessed by the MTT assay 48 h after illumination with 1.8 J/cm^2^. Based on the established IC_50_ values, appropriate concentration ranges for the combination studies were selected for mTHPC and the Pt(II) complexes. Two concentrations below and above the IC_50_ value were used in the MTT viability assay and each of the concentrations for mTHPC or a Pt(II) complex was tested alone or a mTHPC concentration was combined with a concentration of one of the Pt(II) complex with the identical assigned combination number for each cell line. The T/C (treated over control) data from the combination studies were then used for the assessment of synergism and antagonism by the calculation of Combination Indices (CI) by the method of Chou and Talalay [23] (Figure 2). Data presented as means ± SD from at least three independent experiments.

**Figure 2:**
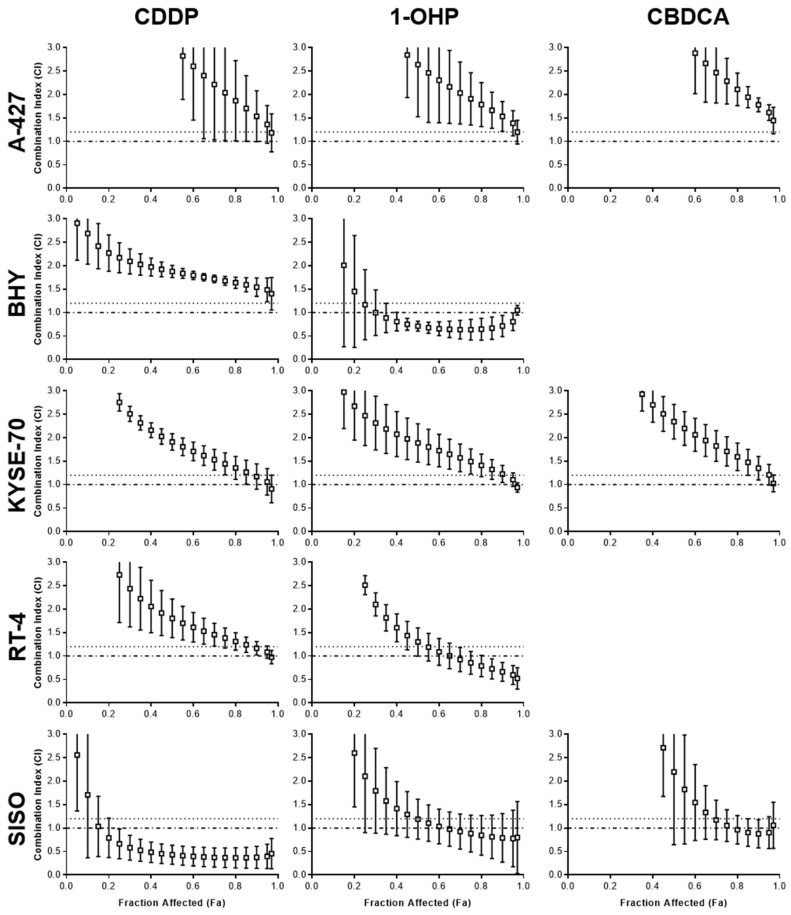
Combination Indices (CI) were calculated from data of MTT assays (Figure 1) and plotted against the fraction affected (Fa) of A-427, BHY, KYSE-70, RT-4 and SISO cells after combination of different concentrations of mTHPC with either CDDP, CBDCA, or 1-OHP for the determination of synergism. CI values below the plotted line at 1.0 indicate synergistic effects by the combined compounds, whereas values > 1.1 indicate antagonistic effects according to the Chou-Talalay method [23].

**Figure A1:**
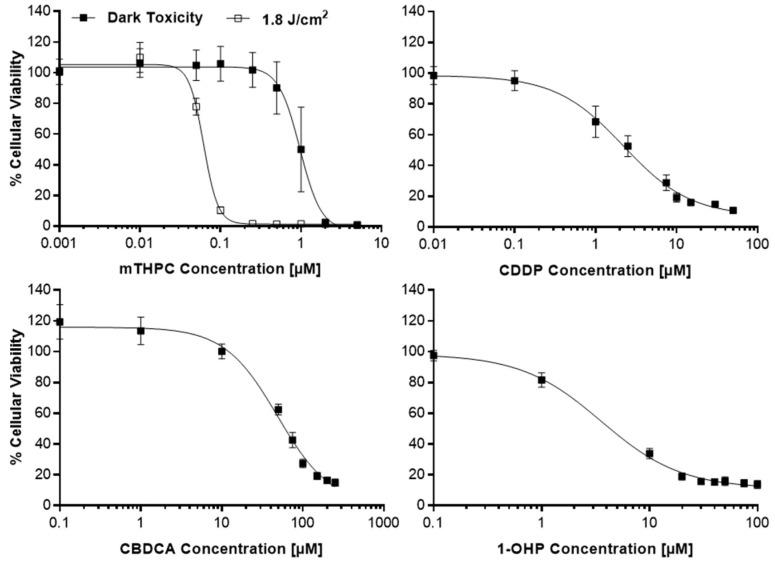
Dark (■) and light-induced (□) loss of cellular viability after mTHPC-mediated PDT or treatment with Pt(II) complexes cisplatin (CDDP), carboplatin (CDBCA) and oxaliplatin (1-OHP) as assessed by the MTT viability assay in SISO cells as a representative sample. Cells were treated with mTHPC for 24 h in concentrations ranging from 0.001–5.0 µM and kept in the dark (dark toxicity) or illuminated with light between *λ* = 640–660 nm and a light fluence of 1.8 J/cm^2^, applied at a fluence rate of 3.0 mW/cm^2^. MTT assay was carried out 24 h post illumination and the absorbance of the reduced formazan was measured at *λ* = 570 nm. The IC_50_ values for the Pt(II) complexes were detected 48 h after treatment without illumination of the cells. Concentrations varied for CDDP (0.01–50.0 µM), CBDCA (0.1–250 µM; 0.001–5.0 mM for A-427) and 1-OHP (0.1–100 µM). The percentage of cell viability was calculated by dividing the absorbance for the treated group by the absorbance in the solvent dark control. IC_50_ values were calculated using Prism 6. Results presented as means ± SD from at least three independent experiments.

Cytotoxic potencies for mTHPC, with and without illumination between *λ* = 640–660 nm at a light fluence of 1.8 J/cm^2^, and the Pt(II) complexes CDDP, CBDCA and 1-OHP were established by the MTT viability assay and set as the inflection points (IC_50_) of the sigmoidal log(dose)-T/C (treated over control) curves (Figure A1 in Appendix A). All IC_50_ values are listed in Table 1. Two concentrations below and two above the IC_50_ value were then selected for each cell line for the subsequent Combination Index (CI) studies by the method of Chou and Talalay [23]. Chou stated that synergistic effects are more than additive effects and synergy or antagonism are mutual effects, other than enhancement, potentiation or augmentation, which are one-sided [23]. The calculation of CI values allows a quantitative definition for additive effects (CI = 1.0), synergism (CI < 1.0) and antagonism (CI > 1.1). In the present studies, each of the concentrations for mTHPC or a Pt(II) complex was tested alone or a mTHPC concentration was combined with a concentration of one of the Pt(II) complex with the identical assigned combination number for each cell line (Figure 1; see Section 4.2 for exact concentrations). The T/C data from the combination studies were then used for the assessment of synergism and antagonism by the calculation of Combination Indices (CI) (Figure 2). No IC_50_ values could be established for CBDCA in BHY and RT-4 cells due to a high toxicity to the solvent DMF (BHY) and a high resistance against CBDCA (RT-4), respectively. Therefore, both cell lines have not been tested in the subsequent combination assay with CBDCA and mTHPC.

Synergistic effects after combination of mTHPC-PDT and Pt(II) complexes were observed in some cases but not in every cell line with all Pt(II) complexes (Figure 2). A combination of mTHPC and CDDP led to slight synergism in KYSE-70 cells at high concentrations and a remarkable synergism in SISO cells at medium and high concentrations (CI < 1.0). However, no synergistic effects were detected with CDDP in RT-4 cells and even antagonistic effects were observed in A-427 and BHY cells. CBDCA together with mTHPC-PDT produced slightly synergistic effects at high concentrations in SISO cells, whereas only antagonistic effects have been detected in A-427 and KYSE-70 cells. The combination of 1-OHP and mTHPC led to synergism at medium concentrations in BHY as well as at medium and high concentrations in RT-4 and SISO cells. No synergistic effects were observed in A-427 and KYSE-70 cells with 1-OHP.

Finally, it should be noted, that no synergism but only antagonism was detected in A-427 cells for any of the tested combinations and, in contrast, all combinations of mTHPC and any of the three tested Pt(II) complexes led to synergistic effects in SISO cells.

For a further analysis of reactive oxygen species (ROS) formation, apoptosis and cell cycle distribution, only selected cell lines and combinations of mTHPC and a Pt(II) complex in promising concentrations that showed synergistic effects in the combination assay were investigated (see Section 4.2 for selected concentrations).

### 2.2. Elevated ROS Levels after Combination of mTHPC-PDT and a Pt(II) Complex

**Figure A2:**
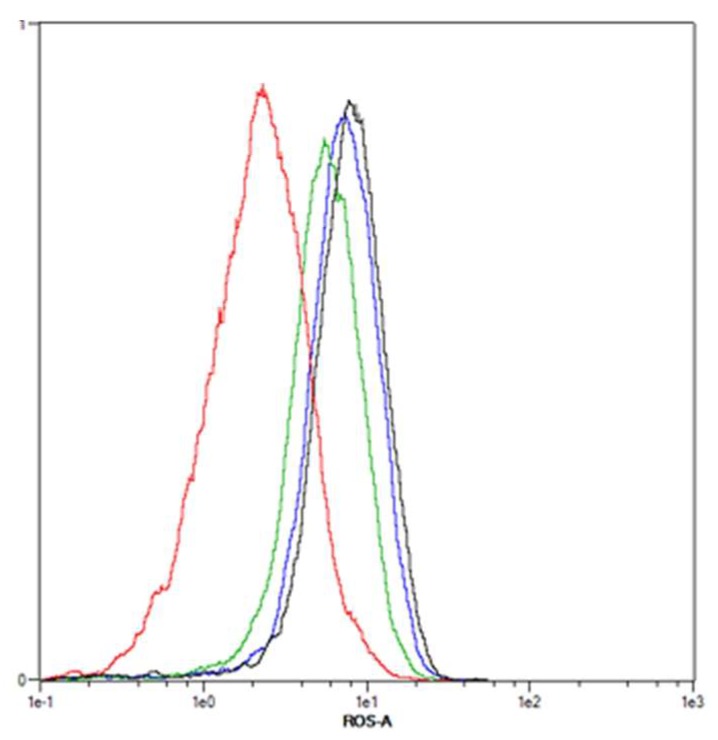
Representative flow cytometric analysis of ROS formation in SISO cells. Cells were treated with solvent in the dark (red) or mTHPC alone (blue), 1-OHP alone (green) or a combination of THPC and 1-OHP (black) prior to illumination with 1.8 J/cm^2^ and stained with H_2_DCF-DA 48 h post illumination. For each sample, 10,000 events were counted and gated for the single cell population (not shown). Fluorescent DCF is formed within the cells after contact with ROS and the detected fluorescence intensity is increased with higher amounts of ROS. The B1 channel (*λ*_Ex/Em_ = 488 nm/525–550 nm) of a MACS Quant flow cytometer was used for the detection of DCF and data were analysed with the MACS Quantify Software.

Increased levels of ROS have been observed after mTHPC treatment [24], which may lead to a sensitization to Pt(II) complexes. In this study, the generation of ROS was detected by flow cytometric analysis of 2′,7′-dichlorofluorescein (DCF) fluorescence intensity (Figure A2 in Appendix A). Fluorescent DCF is formed within the cells after contact with ROS and the detected fluorescence intensity is increased with higher amounts of ROS. Solvent-treated and non-illuminated cells served as a negative reference control (fluorescence intensity for this sample was set to 1.0, not shown).

**Figure 3:**
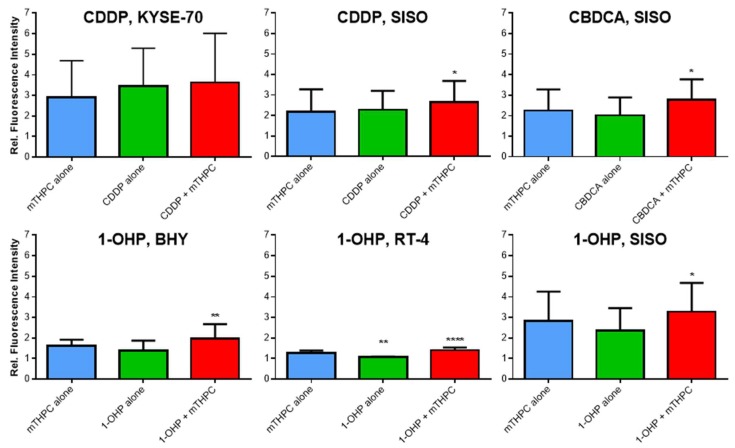
ROS formation after treatment with mTHPC or Pt(II) complex alone or in combination in various cell lines. Cells were illuminated with 1.8 J/cm^2^ after treatment, stained with H_2_DCF-DA and DCF fluorescence intensity was measured by directly after illumination. Cells were gated for the single cell population and flow cytometric analysis was carried out using the B1 channel (*λ*_Ex/Em_ = 488 nm/525–550 nm). Fluorescence intensity was plotted relative to a solvent-treated, non-illuminated negative control (fluorescence intensity set to 1.0, not shown). Data presented as means ± SD from at least three independent experiments. (* *p* < 0.05; ** *p* < 0.01; *** *p* < 0.001; **** *p* < 0.0001). Statistical comparisons were always made between substance treated samples and a solvent-treated, non-illuminated reference control.

Figure 3 shows the results of the intracellular ROS analyses. The dark controls with mTHPC or the combination of mTHPC and either of the three Pt(II) drugs showed no formation of ROS compared to solvent control (data not shown). A combination of mTHPC and CDDP with light led to a significantly higher generation of ROS only in SISO cells. Fluorescence intensity was 2.7-fold increased in this cell line after combination, whereas neither mTHPC nor CDDP alone (2.2- and 2.3-fold increase, respectively) produced significantly enhanced ROS levels. ROS levels after treatment with mTHPC or CDDP alone as well as in combination were even higher in KYSE-70 cells (2.9-fold for mTHPC, 3.5-fold for CDDP and 3.6-fold in combination) but no significant differences were detected compared to the negative, solvent treated control. ROS levels after combination of CBDCA and mTHPC were only analysed in SISO cells. Treatment with either the Pt(II) complex or the PS alone led to 2.0–2.3-fold enhanced ROS levels but a significant difference to the solvent-treated cells was observed after combination of both compounds (2.8-fold increase). A combination of 1-OHP and mTHPC elevated ROS levels in BHY, RT-4 and SISO cells significantly compared to a solvent treated, non-illuminated control. Compared to the reference sample, the formation of ROS was 1.4- and 3.3-fold higher, respectively. Treatment with 1-OHP alone increased ROS levels 1.1–2.4-fold. The generation of ROS after mTHPC-PDT alone was higher (1.6–2.8-fold increase) compared to the Pt(II) complex alone with even significant enhancement in RT-4 cells.

In summary, enhanced ROS levels were observed after combination of mTHPC and CDDP, CBDCA and 1-OHP in all cell lines compared to a treatment with either compound alone but the increase was not significant in all cases.

### 2.3. Combination of mTHPC-PDT and a Pt(II) Complex Can Lead to Enhanced Phosphatidylserine Externalization as a Sign of Increased Apoptosis Induction

**Figure A3:**
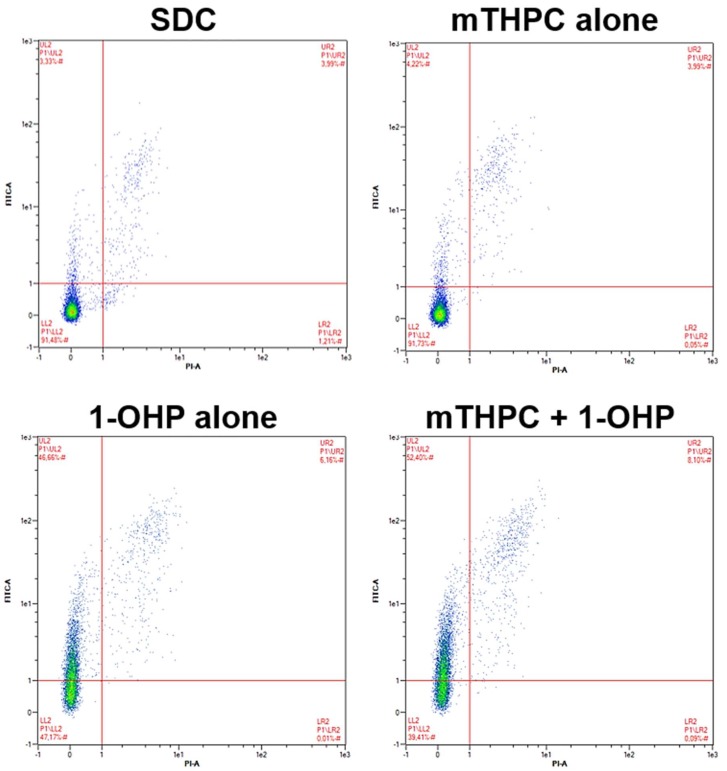
Representative dot plots after flow cytometric analysis of apoptosis induction in SISO cells. Cells were treated with a solvent dark control (SDC), mTHPC or 1-OHP alone, or a combination of mTHPC and 1-OHP prior to illumination with 1.8 J/cm^2^. Double staining with Annexin V-FITC and propidium iodide (PI) was carried out 48 h after illumination. For each sample, 10,000 events were counted and gated for the single cell population (not shown). Unstained, vital cells appear in the lower left quadrant, Annexin V-FITC-positive (apoptotic) cells in the upper left quadrant and Annexin V-FITC- and PI-positive (late-apoptotic/necrotic) cells appear in the upper right part. The B1 channel (*λ*_Ex/Em_ = 488 nm/525–550 nm) of a MACS Quant flow cytometer was used for the detection of Annexin V-FITC and the B3 channel (*λ*_Ex/Em_ = 488 nm/655–730 nm) was used for the PI signal. Data were analysed with the MACS Quantify Software.

The induction of apoptosis has been shown after both mTHPC-PDT [14] and treatment with Pt(II) complexes [7]. However, combination of both treatments may lead to increased apoptotic cell death. In this study, the detection of apoptosis induction has been carried out via the detection of phosphatidylserine at the outer leaflet of the plasma membrane after double staining cells with Annexin V-FITC and PI and subsequent flow cytometric analysis (Figure A3 in Appendix A). Annexin V-FITC selectively stains phosphatidylserine, which is located at the outer leaflet of the cellular membrane after induction of apoptosis [25]. Double staining with PI allows for discrimination of early and late stages of apoptosis, because at later stages the membrane becomes permeable for PI, which then stains nuclear DNA in addition to the staining of phosphatidylserine.

Background levels of phosphatidylserine externalization in the solvent dark control (SDC), which served as the reference sample, were low. In all tested cell lines, 3.1–6.2% of the cells were Annexin V-FITC-positive (apoptotic) and 1.6–2.7% were positive for Annexin V-FITC and PI (late-apoptotic).

**Figure 4:**
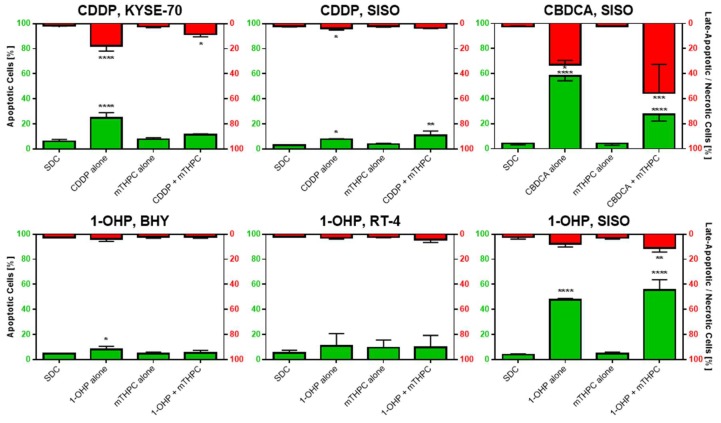
Apoptosis, detected by the Annexin V-FITC/PI method, after treatment with mTHPC-PDT, with a Pt(II) complex alone or after combination of both in various cell lines. Cells were illuminated 24 h after treatment with a light fluence of 1.8 J/cm^2^ and stained with Annexin V-FITC and PI 48 h after illumination. The percentage of apoptotic cells (Annexin V-FITC-positive) is plotted on the left axis and the late-apoptotic cells (Annexin V-FITC- and PI-positive) are shown on the right axis in reversed orientation. Non-illuminated, solvent treated cells (solvent dark control, SDC) were used as the reference sample in statistical analysis. Data presented as means ± SD from at least three independent experiments. (* *p* < 0.05; ** *p* < 0.01; *** *p* < 0.001; **** *p* < 0.0001).

Figure 4 shows the results of the apoptosis analyses. Treatment with CDDP alone produced significantly more apoptotic cells in both KYSE-70 (24.7%) and SISO (7.8%) cells compared to the SDC. Late-apoptotic cells were also significantly increased in both cell lines (KYSE-70: 17.8%; SISO: 3.9%). mTHPC alone did not lead to an induction of apoptosis at the tested concentrations in any cell line (KYSE-70: 7.8%; SISO: 3.9%). Diverging results were obtained in both tested cell lines after combination of mTHPC and CDDP. In KYSE-70, apoptotic cell population was lower (11.3%) compared to the treatment with CDDP alone and not significantly different from SDC (6.2%). Late-apoptotic cell population was significantly increased to 8.5% after combination compared to 1.6% in the negative control but again lower than after treatment with CDDP alone. In SISO, the number of apoptotic cells, however, was significantly higher (10.8%) in comparison to SDC (3.1%) and higher than after treatment with CDDP alone. A fraction of 3.6% of SISO cells were late-apoptotic after combination, which was no difference to the SDC sample (2.9%).

Treatment of SISO cells with CBDCA alone led to a remarkable induction of apoptosis; 58.3% of the cells were assigned to this group, which was significantly higher than in the negative control (4.2%) (Figure 4). Late apoptosis was also very prominent in this sample with 32.9% of the cells being Annexin V-FITC- and PI-positive. After mTHPC-PDT alone, no induction of apoptosis in SISO cells has been observed (4.1%). After combination of mTHPC and CBDCA, a switch to late-apoptotic cells was detected. Apoptotic population dropped to 27.4%, while the late-apoptotic fraction was increased to 55.6%.

Treatment with 1-OHP alone led to similar results in BHY and RT-4 cells, which produced a slight increase in the apoptotic population (BHY: 8.2%; RT-4: 11.0%) compared to the reference (BHY: 4.6%; SISO: 5.3%) (Figure 4). Treatment with mTHPC alone plus light slightly induced apoptosis in RT-4 cells (9.5%) but not in BHY cells (4.8%). After combination of mTHPC and 1-OHP, no changes in the number of apoptotic cells were detected (BHY: 5.3%; RT-4: 10.0%). Late apoptosis was not observed in both tested cell lines under any tested condition. From all above-mentioned samples, the only significant difference to the negative control was observed in BHY cells after treatment with 1-OHP alone. In contrast to that, significantly increased amounts of apoptotic and late-apoptotic cells were found for the SISO cell line. Apoptosis was induced after treatment with 1-OHP alone in 47.8% of the cells and 7.7% of the cells were found to be late-apoptotic. The mTHPC-mediated PDT did not increase the apoptotic population (4.9%) compared to the SDC (4.1%). A combination of mTHPC and 1-OHP led to significantly elevated levels of apoptosis to 55.5% as well as late apoptosis to 10.1% in the cell.

### 2.4. Cell Growth Arrest and DNA Fragmentation Observed in Cell Cycle Analysis

**Figure A4:**
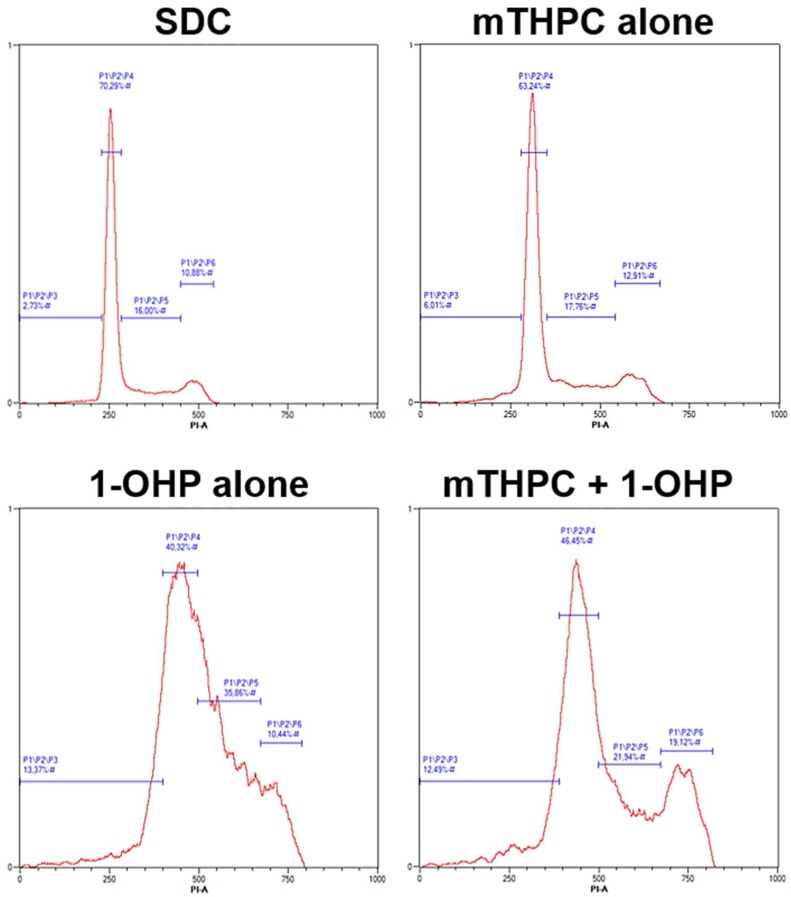
Representative histograms after flow cytometric analysis of cell cycle distribution in SISO cells. Cells were treated with a solvent dark control (SDC), mTHPC or 1-OHP alone, or a combination of mTHPC and 1-OHP prior to illumination with 1.8 J/cm^2^. Fixation and staining with PI were carried out 48 h after illumination. For each sample, 10,000 events were counted and gated for the single cell population (not shown) and cell were assigned to either subG_1_ (fragmented DNA, apoptotic), G_0_/G_1_, S, or G_2_/M phase. The B3 channel (*λ*_Ex/Em_ = 488 nm/655–730 nm) of a MACS Quant flow cytometer. Data were analysed with the MACS Quantify Software.

To investigate the induction of growth arrest or DNA fragmentation, analysis of cell cycle distribution was done by a flow cytometric measurement after PI staining. DNA fragmentation is a hallmark during apoptosis [26] and it is induced by Pt(II) complexes via DNA strand breaks, that also play a role in cell growth inhibition [27,28,29]. Cell cycle analysis allowed to assign the cells to either sub G_1_ (fragmented DNA, apoptotic), G_0_/G_1_, S, or G_2_/M phase populations (Figure A4 in Appendix A).

**Figure 5:**
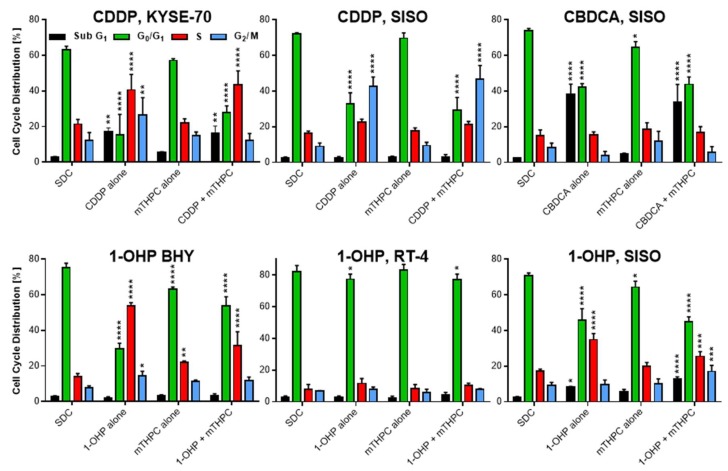
Cell cycle distribution after mTHPC-PDT, treatment with a Pt(II) complex alone or after combination of both treatments in various cell lines. Cells were illuminated 24 h after treatment with a light fluence of 1.8 J/cm^2^ and fixed cells were stained with PI 48 h after illumination. Cells were assigned to either subG_1_ (fragmented DNA, apoptotic), G_0_/G_1_, S, or G_2_/M phase and compared to a non-illuminated, solvent treated sample (solvent dark control, SDC) that served as a reference in statistical analysis. Data presented as means ± SD from at least three independent experiments. (* *p* < 0.05; ** *p* < 0.01; *** *p* < 0.001; **** *p* <0.0001).

Figure 5 shows the results of the cell cycle analyses. Solvent treated, non-illuminated cells showed a cell cycle distribution of 2.4–3.0% in subG_1_, 63.1–82.3% in G_0_/G_1_, 7.9–21.4% in S and 6.9–12.3% in G_2_/M phase. No cell line showed significant differences to this distribution when treated with mTHPC alone (subG_1_: 2.6–5.6%; G_0_/G_1_: 57.2–83.2%; S: 8.2–22.2%; G_2_/M: 6.0–14.9%), except BHY cells, where significantly more cells were found in the S phase of the cell cycle.

After treatment with CDDP alone, significantly more cells with fragmented DNA were found in the subG_1_ population in KYSE-70 (17.3%) but not in SISO cells (2.6%) (Figure 5). Also, S phase cells were significantly higher in KYSE-70 (40.6%) and not in SISO cells (22.6%). However, G_2_/M arrest was induced after treatment with CDDP alone in both cell lines but was higher in SISO cells (KYSE-70: 26.5%; SISO: 42.6%). These results were similar to the results after combination of mTHPC and CDDP. No remarkable changes of the above-mentioned results were observed after combination of mTHPC and CDDP; the only difference was a drop of the G_2_/M fraction in KYSE-70 cells back to normal levels (12.1%) found in the reference control.

CBDCA induced a strong increase in the apoptotic subG_1_ fraction, both after treatment alone (38.3%) and in combination with mTHPC-mediated PDT (33.8%) (Figure 5). No G_2_/M or S phase arrest was detected after treatment, neither with CBDCA alone nor after combination of both compounds.

Treatment with 1-OHP alone did not induce changes in subG_1_ population in BHY (2.1%) and RT-4 cells (3.0%) compared to the SDC sample (Figure 5). In BHY cells, a significant increase in S phase cells to 53.7% as well as in the G_2_/M population to 14.4% was detected after treatment with the Pt(II) complex alone, while G_0_/G_1_ cells dropped to 29.8%. The changes after combination of 1-OHP and mTHPC showed the same trend but were generally lower. S phase and G_2_/M cells were increased to 31.4% and 11.7%, respectively and the G_0_/G_1_ population was decreased only to 53.7%. In RT-4 cells, however, cell cycle distribution was not affected by either 1-OHP alone or in combination with mTHPC in any of the tested samples. In SISO cells, DNA fragmentation was induced after treatment with 1-OHP alone (8.5%) but was even higher after combination with mTHPC (12.6%). Furthermore, a combined treatment led to significantly more cells in the G_2_/M phase (17.0%) compared to no changes after treatment with the Pt(II) complex alone (9.8%). However, S phase population was higher after treatment with 1-OHP alone (34.8%) compared to a combined treatment with mTHPC (25.5%).

## 3. Discussion

The use of Pt(II) complexes in chemotherapy of cancer has an impressive history since the approval of cisplatin (CDDP) in 1978. CDDP has emerged as one of the most successful chemotherapeutic agents for the treatment of solid tumours today; e.g., lung, bladder, cervix and especially testicular cancer. Nevertheless, severe CDDP-related side effects, notably nephrotoxicity and nausea/vomiting, led to the discovery of other promising Pt(II) complexes with improved toxicological profiles, namely carboplatin (CBDCA) and oxaliplatin (1-OHP) [1,6]. Today, CBDCA is used in chemotherapy against ovarian and lung cancer together with paclitaxel (PTX) and 1-OHP treatment is a standard therapy against colorectal cancer. However, chemotherapeutic approaches with these compounds were accompanied by adverse effects as well, mainly myelosuppression (CBDCA) and neurotoxicity (1-OHP) [3,5,30].

All three of the above-mentioned Pt(II) complexes also showed promising results against head and neck cancers [6,18]. Therefore, a combination with 5,10,15,20-tetra(*m*-hydroxyphenyl)chlorin (mTHPC), an approved PS for PDT against head and neck cancers, would appear promising for a combination therapy. Cytotoxic and apoptotic effects on five human cancer cell lines from PDT-relevant and Pt(II)-based chemotherapy-sensitive tissues were evaluated. A combination of both regimens may allow for lower doses of the Pt(II) complex after pre-treatment with and thus enhanced sensitivity towards chemotherapy by mTHPC-mediated PDT without the loss of antitumor efficacy. A combination of mTHPC and chemotherapeutic agents has already been used in studies, where enhanced antitumor effects have been detected with CDDP, doxorubicin, or mitomycin C, both in vitro and in vivo [31,32,33].

We evaluated synergistic effects by calculation of Combination Indices (CI) from MTT viability assay data according to the Chou-Talalay method [23]. Our results showed synergy after combination of mTHPC-mediated PDT and Pt(II) complexes in some selected cases but not in general. In fact, with the A-427 lung cell line, no synergistic but rather strong antagonistic effects were observed after combination of mTHPC with any of the tested Pt(II) complexes, whereas synergy was obtained after combination of mTHPC-PDT with all tested Pt(II) complexes in SISO cervix cells. Antagonistic effects indicate that the antitumor effect after combination is weaker than after treatment with a single compound, which would be highly disadvantageous for therapy. The A-427 lung cell line is the most sensitive cell line against mTHPC with the lowest IC_50_ value among all tested cell lines. However, A-427 cells are the most robust cells against the tested Pt(II) complexes. The opposite is true for SISO cells, that displayed the lowest IC_50_ values for the Pt(II) complexes. The IC_50_ value for mTHPC was in the medium range. These results indicate that a high sensitivity towards the Pt(II) complex seems to be of advantage for an enhancing effect after combination with PDT. The results with the other cell lines confirmed these findings, where always the cell lines with lower IC_50_ values for the Pt(II) complex showed synergism in the combination assay. The only exception was observed with BHY oral cavity cells and CDDP. Although the IC_50_ value against CDDP was similarly low in this cell line compared to the one observed with SISO cells, no synergistic effects were seen after combination with mTHPC. This indicates that cell-specific properties may also play a role for the suitability of a combination therapy and that probably not all cell types are suitable for a combined approach. Biswas and colleagues [34] also used the Chou-Talalay method to identify synergistic effects after treatment of thyroid cancer cells with CBDCA and photosensitizer radachlorin. These authors found synergism at higher concentrations, leading to a significant increase in three hallmarks of apoptosis: phosphatidylserine externalization, caspase 3-activation and poly(ADP-ribose) polymerase (PARP) cleavage. They found evidence that enhanced generation of ROS in the endoplasmic reticulum was responsible for these effects.

The formation of ROS plays a central role in the mechanism of toxicity during PDT [35]. Elevated ROS levels have also been detected after treatment with Pt(II) complexes [18,36]. Therefore, it was of interest whether a combination of mTHPC-PDT and a Pt(II) complex would lead to an enhanced formation of ROS in the treated cells. Except for the combination of CDDP and mTHPC in KYSE-70 oesophageal cells, where only minimal changes of ROS levels were observed after combination, an enhancement of the generation of ROS was observed with 1-OHP in BHY oral cavity and RT-4 bladder cells as well as with CDDP, CBDCA and 1-OHP in SISO cervix cells when compared to a treatment with both mTHPC or a Pt(II) complex alone. Synergistic effects observed in the combination assays are therefore likely induced by an increased generation of ROS within the cells. ROS-mediated sensitization of colorectal cancer cells towards 1-OHP has been shown by Lin et al. [2] after hypericin-mediated PDT. In this study, enhanced apoptotic effects were observed after a combined treatment that was related to a ROS-dependent mechanism. After combination with the ROS scavenger GSH monoethyl ester, the hypericin-induced sensitization towards 1-OHP was attenuated as indicated by decreased induction of apoptosis. Enhanced ROS formation has also been observed by Mao et al. [18] in a study of the combined effects of CBDCA and 9-HPbD-based PDT on laryngeal cancer cells. The authors showed, that both migration and invasion were suppressed more after combination via a ROS-mediated mechanism and concluded, that a combination regimen might be a promising approach for laryngeal cancer metastasis.

Elevated ROS levels have the potential to increase cytotoxic effects due to oxidative stress, which can lead to enhancement of apoptosis. In this study, the Pt(II) complexes alone induced phosphatidylserine externalization in all tested cell lines, as measured by the Annexin V-FITC/PI assay. The induction of apoptosis after treatment with Pt(II) complexes is known and has been shown for CDDP, CBDCA and 1-OHP [28,37,38]. Treatment with mTHPC alone plus light did not lead to an induction of apoptosis in any cell line. This was not surprising, because the applied concentrations of 0.01–0.05 µM were all below the established IC_50_ values (0.06–0.1 µM). After combination of mTHPC and a Pt(II) complex, no enhanced apoptotic effects were observed with CDDP in KYSE-70 oesophageal cells. Likewise, no increased ROS levels have been observed in this cell line. For KYSE-70, a combination of higher concentrations seems to be more suitable, which would be worth investigating in more detail. However, no increased Annexin V-FITC binding has been observed in BHY oral cavity and RT-4 bladder cells as well after combination of mTHPC-PDT with 1-OHP, although more ROS were detected under the same conditions. These results indicate, that the cells were able to compensate higher oxidative stress in some way. In a study with 1-OHP in colon cells, Tan et al. [39] showed that oxaliplatin-induced autophagy reversed its apoptotic effects, which did not happen in the presence of an autophagy inhibitor. A good correlation of elevated ROS levels and increased apoptosis induction has been obtained with a combination of mTHPC-PDT and any of the tested Pt(II) complexes in SISO cervical cells. Apoptosis was higher after combination with mTHPC compared to the treatment with any Pt(II) complex alone. With CBDCA, a switch to late apoptosis was observed after combination.

Cell cycle analysis revealed a block of growth inhibition and DNA fragmentation after treatment with Pt(II) complexes alone, whereas no changes in cell cycle distribution were observed after treatment with mTHPC-PDT except a mild S phase arrest in BHY cells. This, again, was not surprising due to the applied mTHPC concentrations were below the IC_50_ values. CDDP treatment prevented mitosis of the cells via G_2_/M arrest in both KYSE-70 oesophageal and SISO cervical cells. Although CDDP is known for inducing apoptosis as well as S phase and G_2_/M arrests [40,41], S phase arrest and an apoptotic subG_1_ population with fragmented DNA were only observed in KYSE-70 but not in SISO cells. However, apoptosis induction by CDDP in SISO cervical cells was shown via phosphatidylserine externalization, which is an earlier event in the apoptotic cascade [42]. After combination of CDDP and mTHPC-PDT, no changes in cell cycle distribution were obtained for SISO cervical cells. G_0_/G_1_ cells were significantly lower and a prominent G_2_/M arrest was detected similar to the treatment with CDDP alone. In KYSE-70 oesophageal cells, the apoptotic population was comparable to the treatment with CDDP alone, which has not been observed via Annexin V-FITC staining. Furthermore, the G_2_/M arrest was reversed back to control levels after combination of CDDP and mTHPC. Again, a combination of higher concentrations seems appropriate for CDDP and mTHPC in KYSE-70 oesophageal cells. Treatment with CBDCA alone induced a massive subG_1_ population with fragmented DNA equal to the detected apoptosis induction via Annexin V-FITC in SISO cervical cells. No S or G_2_/M arrests were observed, although that has been the case in other studies but for time points later than 24 h [41,43]. No changes have been detected after combination of CBDCA with mTHPC-mediated PDT. These results confirmed the results for CBDCA and mTHPC-PDT in SISO cervical cells observed for phosphatidylserine externalization. No apoptotic subG_1_ populations were detected after treatment with 1-OHP alone in BHY oral cavity and RT-4 bladder cells, confirming the results observed after Annexin V-FITC staining, where it was concluded that both cell lines may compensate ROS-induced stress signals. However, both S and G_2_/M arrests were observed in BHY cells for 1-OHP alone, which has been detected after treatment with 1-OHP before [28,44,45]. Both, S phase and G_2_/M arrested cells were decreased after prior sensitization towards 1-OHP via mTHPC-PDT. A good correlation between phosphatidylserine externalization and cell cycle changes were observed for 1-OHP in SISO cervical cells. Populations of cells with fragmented DNA were obtained after treatment with 1-OHP alone and after combination with mTHPC but were higher for the latter. Furthermore, a comparable S phase arrest was found under both conditions, whereas G_2_/M arrest was observed only after combination of 1-OHP with mTHPC-PDT.

## 4. Materials and Methods

### 4.1. Cell Culture

The five different cell lines A-427 (lung carcinoma; ACC 234), BHY (oral squamous cell carcinoma; ACC 404), KYSE-70 (oesophageal squamous cell carcinoma; ACC 363), RT-4 (urinary bladder transitional cell carcinoma; ACC 412) and SISO (cervix adenocarcinoma; ACC 327) were obtained from DSMZ (Braunschweig, Germany) and cultivated in RPMI 1640 medium (PAN Biotech, Aidenbach, Germany) supplemented with 10% (*v*/*v*) foetal bovine serum (FBS; Sigma-Aldrich, Munich, Germany), 100 µg/mL streptomycin and 100 U/mL penicillin G (PAN Biotech, Aidenbach, Germany) at 37 °C and 5% CO_2_ in a humidified atmosphere. Phenol red free medium was used during and post illumination. Cells were subcultured once a week with a 0.5 g trypsin/0.2 g EDTA solution (Sigma-Aldrich) and seeded in transparent, flat-bottom 96-well plates at a density of 2.0–5.0 × 10^3^ cells per well in 100 µL medium for the measurement of cellular viability, in T25 flasks at a density of 5.0 × 10^5^ cells in 5 mL medium for the analysis of apoptosis and cell cycle distribution and in 6-well plates at a density of 2.5 × 10^5^ cells per well in 2 mL medium for the detection of reactive oxygen species (ROS) (culture vessels from Sarstedt, Nümbrecht, Germany). After seeding, cells were incubated for 24 h before treatment.

### 4.2. Treatment with Photosensitizer and Pt(II) Complexes

The photosensitizer (PS) 5,10,15,20-tetra(*m*-hydroxyphenyl)chlorin (mTHPC, Temoporfin) was kindly provided by Biolitec AG (Jena, Germany). A 20 mM stock solution was prepared in propylene glycol/ethanol (60:40) and stored at 4 °C. For the establishment of IC_50_ values, mTHPC was given to the cells in 96-well plates in different concentrations (0.001–5.0 µM) in 100 µL medium supplemented with 10% (*v*/*v*) FBS for 24 h. Medium was changed to phenol red free medium and the cells were then illuminated with a light fluence of 1.8 J/cm^2^. Cellular viability was analysed 24 h post illumination using the MTT (3-(4,5-dimethyl-2-thiazolyl)-2,5-diphenyl-2*H*-tetrazolium bromide; Alfa Aesar, Karlsruhe, Germany) viability assay. The IC_50_ values for the Pt(II) complexes were measured 48 h after treatment without illumination of the cells. Pt(II) complexes were either cisplatin (*cis*-diamminedichloridoplatinum(II), CDDP; Alfa Aesar, Karlsruhe, Germany), carboplatin (*cis*-diammine-1,1-cyclobutanedicarboxylatoplatinum(II), CBDCA; Glentham, Edinburgh, UK), or oxaliplatin (*trans*-*R*,*R*-cyclohexane-1,2-diamineoxalatoplatinum(II), 1-OHP; Glentham, Edinburgh, UK). For CDDP and 1-OHP, stock solutions were prepared in DMF (50 mM and 10 mM, respectively), whereas sterile H_2_O served as the solvent for CBDCA (10 mM). Concentrations varied for CDDP (0.01–50.0 µM), CBDCA (0.1–250 µM; 0.001–5.0 mM for A-427) and 1-OHP (0.1–100 µM). Based on the established IC_50_, appropriate concentration ranges for the combination studies were selected for mTHPC and the Pt(II) complexes. The PS was added to all cell lines in the same concentrations of 0.001–0.1 µM in 100 µL medium supplemented with 10% (*v*/*v*) FBS and left for 20 h. Afterwards, the Pt(II) complex was given to both mTHPC treated (combination) and solvent treated cells (Pt(II) complex alone) for 4 h before illumination in 100 µL fresh, phenol red free medium. For the assessment of the effects of the PS alone, the mTHPC containing medium was replaced by fresh medium without a Pt(II) complex (mTHPC alone). The concentration ranges for the Pt(II) complexes varied with cell lines and are listed in Table 2. For each cell line, each of the concentration for mTHPC or a Pt(II) complex was tested alone or a mTHPC concentration was combined with one of the concentrations of a Pt(II) complex with the identical assigned combination number. Cellular viability was measured 48 h after illumination of the cells by the MTT assay.

The viability data from the combination studies were then used for the assessment of synergism and antagonism by the calculation of Combination Indices (CI) with the CompuSyn software (ComboSyn, Paramus, NJ, USA) as described by Chou [23,46]. For subsequent analyses of ROS formation (in 6-well plates), phosphatidylserine externalization and cell cycle distribution in (in T25 flasks), only selected cell lines as well as promising concentrations for mTHPC and a corresponding Pt(II) complex were chosen depending on the outcome of the combination assay (Table 3).

### 4.3. Photodynamic Treatment

After the treatment with mTHPC for 20 h and following 4 h incubation period with a Pt(II) complex, cells were washed with phosphate buffered saline (PBS) and fresh, phenol red free RPMI 1640 medium containing 10% (*v*/*v*) FBS was added before illumination. Cells were illuminated using an LED (light-emitting diode) array based illumination device [47]. The LEDs (Kingbright, Issum, Germany) produced a wavelength spectrum of *λ* = 640–660 nm and a light fluence of 1.8 J/cm^2^ was applied at a fluence rate of 3.0 mW/cm^2^. Cells were harvested by trypsinization directly after illumination (ROS formation) or 48 h post illumination (phosphatidylserine externalization, cell cycle distribution) for flow cytometric analyses. Non-illuminated cells treated with solvent in medium only were used as a negative reference control in all assays.

### 4.4. Analysis of Cell Viability by the MTT Assay

At 48 h after illumination, 20 µL of a 2.5 mg/mL MTT solution were added to each 100 µL medium per 96-well and the cells further incubated at 37 °C and 5% CO_2_ for 4 h. Supernatant was replaced afterwards with 50 µL DMSO per well and the absorbance of the reduced formazan was measured at *λ* = 570 nm with a microplate reader (SpectraMax Plus 384; Molecular Devices, Biberach, Germany). The percentage of cell viability was calculated by dividing the absorbance in the treated group by the absorbance in the solvent control.

### 4.5. Analysis of ROS Generation

The detection of generated ROS was analysed after treatment with H_2_DCF-DA (Sigma-Aldrich). Cells were pre-incubated with 2 mL per 6-well of a 20 µM H_2_DCF-DA solution (in PBS) for 30 min at 37 °C and 5% CO_2_. The solution was replaced by fresh PBS and the cells were illuminated at 1.8 J/cm^2^ afterwards. The cells were then harvested directly after photodynamic treatment, washed twice with PBS and flow cytometric analysis of the fluorescent DCF was done with a MACS Quant flow cytometer (Miltenyi Biotech, Bergisch Gladbach, Germany). For each sample, 10,000 events were counted and gated for the single cell population. Fluorescent DCF is formed within the cells after contact with ROS and the detected fluorescence intensity is increased with higher amounts of ROS. The B1 channel (λ_Ex/Em_ = 488 nm/525–550 nm) was used for the detection of DCF (see Figure A2 for representative analysis data) and data were analysed with the MACS Quantify Software (Miltenyi Biotech, Bergisch Gladbach, Germany).

### 4.6. Analysis of Apoptosis by the Annexin V-FITC/Propidium Iodide Assay

To assess for apoptosis, phosphatidylserine membrane externalization was detected by using the Annexin V-FITC kit (Miltenyi Biotech, Teterow, Germany) according to the kit instructions. Briefly, 5.0 × 10^5^ cells were harvested by trypsinization 48 h after photodynamic treatment and cells were washed with Binding Buffer and stained with Annexin V-FITC at room temperature for 15 min in the dark. Cells were washed again and propidium iodide (PI) was added immediately before flow cytometric analysis using the MACS Quant flow cytometer. For each sample, 10,000 events were counted and gated for the single cell population. Annexin V-FITC selectively stains phosphatidylserine, which is typically located at the outer membrane after induction of apoptosis [25]. Double staining with PI allows for discrimination of early and late stages during apoptosis. At later stages, the membrane becomes permeable for PI, which then stains nuclear DNA in addition to the staining of phosphatidylserine. The B1 channel (*λ*_Ex/Em_ = 488 nm/525–550 nm) was used for the detection of Annexin V-positive cells, PI-positive cells were detected with the B3 channel (*λ*_Ex/Em_ = 488 nm/655–730 nm). Data were analysed with the MACS Quantify Software (see Figure A3 for representative analysis data).

### 4.7. Cell Cycle Analysis

Changes in the cell cycle distribution was analysed after staining with PI (AppliChem, Darmstadt, Germany). Cells were treated and 5.0 × 10^5^ harvested 48 h after photodynamic treatment as described for the analysis of phosphatidylserine externalization. Cells were washed twice with PBS and fixed by ice-cold 70% (*v*/*v*) ethanol at 4 °C for 30 min. Fixed cells were centrifuged at 4.000 rpm at 4 °C for 10 min and resuspended in PBS containing 25 µg/mL PI and 100 µg/mL RNase A (Carl Roth, Karlsruhe, Germany). After staining at room temperature for 30 min in the dark, cells were analysed with a MACS Quant flow cytometer. For each sample, 10,000 events were counted and gated for the single cell population. The B3 channel (λ_Ex/Em_ = 488 nm/655–730 nm) was used for the detection of PI-positive cells. Data were analysed with the MACS Quantify Software. During analysis, cells were assigned to either sub G_1_ (fragmented DNA, apoptotic), G_0_/G_1_, S, or G_2_/M phase populations (see Figure A4 for representative analysis data).

### 4.8. Statistical Analysis

Data were presented as means ± standard deviation (SD) of at least three independent experiments. Significant differences were detected by one-way or two-way ANOVA followed by Dunnett’s multiple comparisons test implemented by Prism 6 (GraphPad Software, La Jolla, CA, USA). A *p* value < 0.05 was considered statistically significant. Comparisons were always made between substance treated samples and solvent-treated controls.

## 5. Conclusions

In conclusion, pre-treatment with mTHPC-mediated PDT has the potential to sensitize cells towards the most widely used Pt(II) complexes CDDP, CBDCA and 1-OHP, although differences were detected among cell lines and with varying Pt(II) complexes. A-427 lung cells did not show any synergistic effects after a combined treatment, whereas SISO cervical cells were suitable for a combination with all tested Pt(II) complexes. In BHY oral cavity, KYSE-70 oesophageal and RT-4 bladder cells, synergistic effects were obtained only for one of the tested Pt(II) complexes. 1-OHP showed higher synergism in the tested cell lines compared to CDDP and CBDCA. Elevated ROS levels were detected after combination that seem to be responsible for the induction of apoptosis. However, an increased induction of apoptosis was not detected after combination of mTHPC and a Pt(II) complex under all tested conditions. Apoptotic cell death was mainly accompanied by cell cycle arrests in S and G_2_/M phase and the appearance of subG_1_ populations with fragmented DNA confirmed the induction of apoptosis. Finally, our results demonstrate that a combined treatment approach of mTHPC-PDT and Pt(II) complexes has to be evaluated individually for each specific cancer type, because combinations do not always lead to increases in the antitumor efficacy and can even be antagonistic.

## Figures and Tables

**Table 1:** Calculated IC_50_ values from MTT viability assay for mTHPC + light and the Pt(II) complexes CDDP, CBDCA and 1-OHP in A-427, BHY, KYSE-70, RT-4 and SISO cells.

Substance	IC_50_ ± SD (µM)				
	A-427	BHY	KYSE-70	RT-4	SISO
**mTHPC + light**	0.02 ± 0.01	0.06 ± 0.01	0.06 ± 0.01	0.10 ± 0.01	0.06 ± 0.002
**Cisplatin**	17.3 ± 0.80	3.22 ± 0.63	8.15 ± 0.99	12.9 ± 6.76	2.31 ± 0.89
**Carboplatin**	205 ± 39.0	n.d.	220 ± 31.0	n.d.	50.1 ± 2.09
**Oxaliplatin**	45.4 ± 10.7	27.1 ± 3.74	36.7 ± 7.50	11.7 ± 4.73	3.41 ± 0.42

n.d.—not determined.

**Table 2:** Cell-specific concentrations of mTHPC and Pt(II) complexes as used in the combination assay. For each cell line, each of the concentration for mTHPC and CDDP, CBDCA, or 1-OHP was tested alone or the concentrations for a Pt(II) complex were combined with a mTHPC concentration with the identical assigned combination number.

**Comb.**	**Cisplatin ** **(** **µM)**					**Comb.**	**Carboplatin (µM)**		
**Number**	**A-427**	**BHY**	**KYSE-70**	**RT-4**	**SISO**	**Number**	**A-427**	**KYSE-70**	**SISO**
**1**	5.0	1.00	1.00	1.00	0.10	**1**	50.0	50.0	10.0
**2**	10.0	2.00	5.00	5.00	0.50	**2**	100	100	50.0
**3**	15.0	4.00	10.0	10.0	1.00	**3**	200	200	100
**4**	20.0	6.00	15.0	15.0	2.50	**4**	300	300	150
**5**	30.0	8.00	20.0	20.0	5.00	**5**	100	400	200
**Comb.**	**Oxaliplatin (µM)**					**Comb.**	**mTHPC (µM)**		
**Number**	**A-427**	**BHY**	**KYSE-70**	**RT-4**	**SISO**	**Number**	**All Cell Lines**		
**1**	10.0	1.00	10.0	1.00	1.00	**1**	0.001		
**2**	25.0	10.0	25.0	5.00	5.00	**2**	0.010		
**3**	50.0	25.0	50.0	10.0	7.50	**3**	0.025		
**4**	75.0	50.0	75.0	15.0	10.0	**4**	0.050		
**5**	100	75.0	100	25.0	15.0	**5**	0.100		

**Table 3:** Concentrations of mTHPC and Pt(II) complexes as combined in further analyses of ROS formation, phosphatidylserine externalization and cell cycle distribution. Only selected cell lines as well as promising concentrations for mTHPC and a corresponding Pt(II) complex were chosen depending on the outcome of the combination assay.

Cell Line	mTHPC (µM)	Pt(II) Complex (µM)		
		CDDP	CBDCA	1-OHP
**A-427**	n.t.	n.t.	n.t.	n.t.
**BHY**	0.025	n.t.	n.t.	25.0
**KYSE-70**	0.025	10.0	n.t.	n.t.
**RT-4**	0.050	n.t.	n.t.	15.0
**SISO**	0.010; 0.025 ^#^	0.50	100	7.5

n.t.—not tested in further analyses. ^#^ 0.010 µM mTHPC combined with CDDP, 0.025 µM mTHPC combined with CBDCA and 1-OH.

## Abbreviations

(N)SCLC	(Non-)Small Cell Lung Cancer
1-OHP	Oxaliplatin, *trans*-*R,R*-Cyclohexane-1,2-diamineoxalatoplatinum(II)
9-HPbD	9-Hydroxypheophorbide α
CBDCA	Carboplatin, *cis*-Diammine-1,1-cyclobutanedicarboxylatoplatinum(II)
CDDP	Cisplatin, *cis*-Diamminedichloridoplatinum(II)
CI	Combination Index
DCF	2′,7′-Dichlorofluorescein
Fa	Fraction affected
FBS	Fetal Bovine Serum
GSH	Glutathione
H_2_DCF-DA	2′,7′-Dichlorodihydrofluorescein Diacetate
LED	Light-emitting Diode
mTHPC	5,10,15,20-tetra(*m*-Hydroxyphenyl)chlorin
MTT	3-(4,5-Dimethyl-2-thiazolyl)-2,5-diphenyl-2*H*-tetrazolium Bromide
PARP	Poly(ADP-Ribose) Polymerase
PBS	Phosphate Buffered Saline
PDT	Photodynamic Therapy
PI	Propidium Iodide
PTX	Paclitaxel
ROS	Reactive Oxygen Species
SDC	Solvent Dark Control
